# Vector competence of *Aedes aegypti* from Havana, Cuba, for dengue virus type 1, chikungunya, and Zika viruses

**DOI:** 10.1371/journal.pntd.0008941

**Published:** 2020-12-03

**Authors:** Gladys Gutiérrez-Bugallo, Antoine Boullis, Yanet Martinez, Lyza Hery, Magdalena Rodríguez, Juan A. Bisset, Anubis Vega-Rúa

**Affiliations:** 1 Department of Vector Control, Center for Research, Diagnostic, and Reference, Institute of Tropical Medicine Pedro Kourí, PAHO-WHO Collaborating Center for Dengue and its Control, Havana, Cuba; 2 Institut Pasteur of Guadeloupe, Laboratory of Vector Control research, Unit Transmission Reservoir and Pathogens Diversity, Les Abymes, France; INDEPENDENT RESEARCHER, UNITED STATES

## Abstract

**Background:**

Like many countries from the Americas, Cuba is threatened by *Aedes aegypti*-associated arboviruses such as dengue (DENV), Zika (ZIKV), and chikungunya (CHIKV) viruses. Curiously, when CHIKV was actively circulating in the region in 2013–2014, no autochthonous transmission of this virus was detected in Havana, Cuba, despite the importation of chikungunya cases into this city. To investigate if the transmission ability of local mosquito populations could explain this epidemiological scenario, we evaluated for the first time the vector competence of two *Ae*. *aegypti* populations (Pasteur and Párraga) collected from Havana for dengue virus type 1 (DENV-1), CHIKV, and ZIKV.

**Methodology/Principal findings:**

Mosquito populations were fed separately using blood containing ZIKV, DENV-1, or CHIKV. Infection, dissemination, and transmission rates, were estimated at 3 (exclusively for CHIKV), 7, and 14 days post exposure (dpe) for each *Ae*. *aegypti* population-virus combination. Both mosquito populations were susceptible to DENV-1 and ZIKV, with viral infection and dissemination rates ranging from 24–97% and 6–67% respectively. In addition, CHIKV disseminated in both populations and was subsequently transmitted. Transmission rates were low (<30%) regardless of the mosquito population/virus combination and no ZIKV was detected in saliva of females from the Pasteur population at any dpe.

**Conclusions/Significance:**

Our study demonstrated the ability of *Ae*. *aegypti* from Cuba to transmit DENV, ZIKV, and CHIKV. These results, along with the widespread distribution and high abundance of this species in the urban settings throughout the island, highlight the importance of *Ae*. *aegypti* control and arbovirus surveillance to prevent future outbreaks.

## Author summary

Dengue (DENV), Zika (ZIKV), and chikungunya (CHIKV) viruses are mainly transmitted by *Aedes aegypti* mosquitoes and together caused near 10 million human cases in the last decade in the Americas. The diseases caused by these viruses share symptoms ranging from mild clinical manifestations to severe complications, including fatal outcomes. Cuba has not been exempted from these viruses and is frequently subjected to dengue and more recently by diseases caused by ZIKV infection. Surprisingly, despite multiple CHIKV outbreaks recorded in neighboring countries, local circulation has not been reported in Havana. Although *Ae*. *aegypti* has been implicated as the major DENV and ZIKV vector because of its wide distribution on the island, neither field nor experimental approaches have been used to demonstrate the ability of Cuban *Ae*. *aegypti* populations to transmit DENVs, ZIKV, and CHIKV. In this laboratory study, we demonstrated that two *Ae*. *aegypti* populations from Havana could become infected with and transmit these viruses, albeit at low rates. Our paper suggests that the absence of CHIKV circulation in Havana was not related to refractoriness of *Ae*. *aegypti* populations to this virus; therefore, vector control remains being essential in preventing future arboviruses outbreaks.

## Introduction

Since 2010, the Americas have been facing increased emergence and re-emergence of viral agents transmitted by *Aedes* mosquitoes, raising global concerns about their public health consequences as well as the feasibility of their prevention and control. Between 2013 and 2017, the American countries notified around 9.8 million clinical cases of disease associated with infection with dengue (DENV), chikungunya (CHIKV) and Zika (ZIKV) viruses combined [1]. These viruses are active in Cuba, with the country reporting several dengue outbreaks since 1977 [2]. More recently, public health authorities reported outbreaks with co-circulation of DENV and ZIKV viruses affecting all the provinces with 10,162 and 2,633 reported cases, respectively between 2015 and 2019 [1]. Despite importation of around 200 CHIKV cases from neighboring regions of the Americas [1] and the detection of autochthonous transmission in the eastern province of Santiago de Cuba [2], transmission of this virus has not been reported in Havana.

CHIKV, DENV, and ZIKV are arboviruses that have similar epidemiology, share the same vector species, and the epidemics they cause are mainly associated with their circulation in urban transmission cycles [3]. Dengue is a complex of viruses that includes four genetically and antigenically different serotypes (DENV-1–4) [4], as well as multiple genetic lineages of each serotype [5]. DENV and ZIKV are flaviviruses (family *Flaviviridae*) with a single-stranded 11 kb RNA genome that share common epitopes on the envelope protein that can cross-react in serological tests [6]. ZIKV was first detected in East Africa in 1947 [7]. Three ZIKV linages have been recognized: African, Asian, and American. The latter two have been associated with neurological disorders such as congenital Zika syndrome and Guillain Barré syndrome [8]. CHIKV is an alphavirus (family *Togaviridae*) that was first described in Tanzania in 1952 during an epidemic of dengue-like illness [9]. According to their genetic diversity, three major genotypes of CHIKV have been identified: West African, East/Central/South African (ECSA), and Asian [10]. Since 2013, the Asian linage of CHIKV has circulated in several Caribbean, South-, and Central-American countries, causing >1 million cases in 2014 [1].

It has been demonstrated that in urban environments in the Americas, these three arboviruses are mainly transmitted by *Aedes (Stegomya) aegypti* mosquitoes [11]. In the absence of effective vaccines or specific treatment against these viruses, public health efforts must rely in the control of the vector populations so knowledge of their biology and ecology are essential in tackling the transmission. For instance, understanding the capacity of local mosquito species to transmit medically important pathogens is crucial for assessing risk and for targeting control programs. The vector competence of a given mosquito species is defined as its intrinsic ability to become infected after the ingestion of an infectious blood meal and subsequently support replication, dissemination, and the transmission of the pathogen to a new susceptible host via the infected saliva delivered during a blood meal [12]. Vector competence is variable and highly dependent upon the mosquito population, pathogen strain and their genotype-by-genotype interactions [13].

In Cuba, *Ae*. *aegypti* has been implicated as the major DENV and ZIKV vector due to its abundance and wide distribution in domestic settings [14]. Nevertheless, neither field nor experimental approaches have been used to demonstrate the ability of Cuban *Ae*. *aegypti* populations to transmit DENV, ZIKV, and CHIKV. Herein, we assessed for the first time the vector competence of two *Ae*. *aegypti* populations from Cuba for dengue virus type 1 (DENV-1), ZIKV, and CHIKV to (i) characterize the infection, dissemination, and transmission ability of these mosquito populations for the three viruses, and (ii) explore whether the absence of autochthonous CHIKV transmission in Havana city could be linked to limited vector competence of local *Ae*. *aegypti* populations to this virus.

## Methods

### Ethics statement

This study has been approved by the internal ethics committee of the Pasteur Institute of Guadeloupe (established since September 2015). Anubis Vega-Rúa (author of the study) provided written consent for blood donation to artificially feed mosquitoes in experiments.

### Mosquito populations

**Fig 1:**
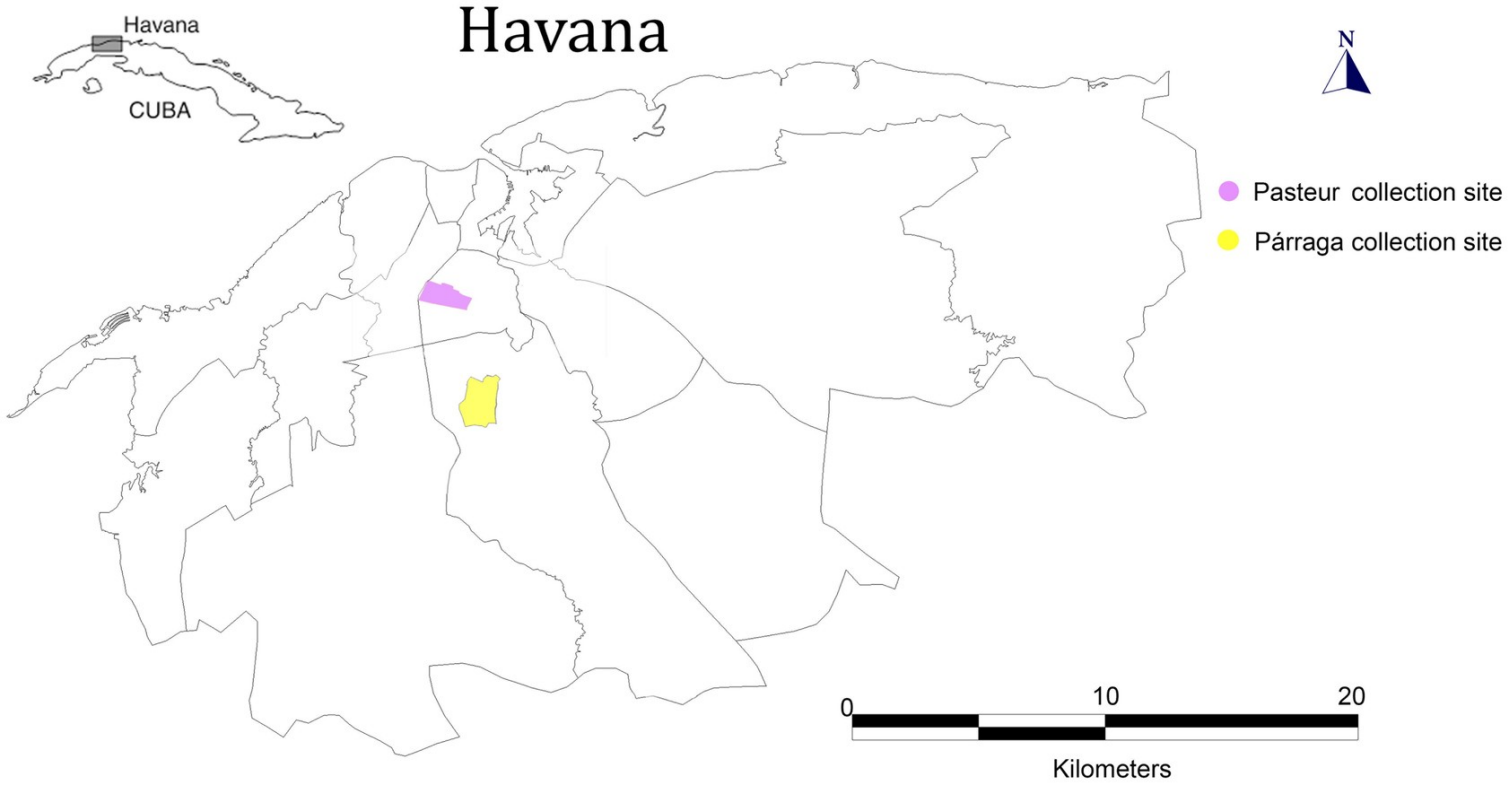
Havana province. Divisions inside the province indicate each Havana’s municipality. Colored areas show the *Aedes aegypti* collection locations. The area in purple corresponds to Pasteur site and the area in yellow corresponds to Párraga site.

Two *Ae*. *aegypti* populations were collected using ovitraps in Havana, Cuba at the municipal health areas of Pasteur (PTE) (23.094494, 82.365780) and Párraga (PRG) (23.06199, 82.353201) that belong to “Diez de Octubre” and “Arroyo Naranjo” municipalities respectively, and which are separated by 7 km (Fig 1). These locations were classified as low (PTE) and high (PRG) flavivirus transmission risk areas by the health authorities based on human case records during 2009–2017.

Eggs were hatched in dechlorinated tap water and larvae were reared under controlled conditions (28°±1°C with a 16h:8h light:dark cycle, 80% relative humidity) at a density of 150–200 larvae/liter and fed on yeast tablet every 3–4 days. Adults were kept in cages under the same conditions described above and supplied *ad libitum* with a 10% sucrose solution. An artificial blood meal was provided using a Hemotek system (Hemotek Ltd., Blackburn, UK) to obtain the F_1_ generation of mosquitoes used in this study.

To assess the absence of arboviral infection in the F_1_ eggs used, the infectious status of the F_0_ adults derived from collected eggs (900) was determined by real time RT-PCR with the kits *Lightmix Modular Zika* (Roche, Panama) and Lightmix *Modular Dengue* (Roche, Panama), according to manufacturer’s instructions. None of these adults were infected, suggesting that mosquitoes used in this study were from uninfected eggs.

### Viral strains

The DENV-1, CHIKV, and ZIKV strains were isolated from the sera of human cases that were detected in Guadeloupe and confirmed by RT-PCR in 2013, 2014, and 2016, respectively. Partial sequencing of CHIKV NS1 gene (Accession number: LR792670.1) revealed 97.7% identity with a strain from Suriname (Accession number: KY435463.1) isolated during CHIKV outbreaks in 2014, while that of NS5 ZIKV gene (Accession number: LR792671.1) showed 92.96% identity with a strain isolated in Colombia during the 2016 outbreaks (Accession number: MK049249.1). The partial DENV-1 sequence was obtained from E gene (Accession number: LR792669.1) and showed 96.2% identity with a strain isolated in Haiti in 2012 (Accession number: MG877553.1).

Virus stocks were generated using a multiplicity of infection of 0.1, after two passages on African green monkey kidney Vero cells (ATCC, ref. CCL- 81) for CHIKV and ZIKV, and two passages on C6/36 *Aedes albopictus* cells for DENV-1. Supernatants were collected and the viral titer was estimated by cytopathic effect (CPE) examination using serial 10-fold dilutions on Vero cells expressed as median tissue culture infectious dose (TCID_50_/mL) for CHIKV and ZIKV. Because DENV does not always produce CPE in mammal cells, its viral titer was estimated by focus fluorescent assay on *Ae*. *albopictus* C6/36 cells and expressed in focus-forming units (FFU/mL). The virus stocks were stored at -80°C until use.

### Experimental mosquito exposures

Mosquito exposures were conducted with a Hemotek system, using pig intestine membranes. Seven- to 10-day-old female mosquitoes were fed on an infectious blood-meal containing 1.4 mL of washed heparin-treated human erythrocytes, 700 μL of virus suspension at desired concentration, and supplemented with a phagostimulant (ATP), provided at a final concentration of 5 mM. For each mosquito population, three groups of 60 female mosquitoes were exposed to each viral strain (ZIKV, DENV-1, and CHIKV). The titers of infectious blood meals were 10^7^ TCID_50_/mL for CHIKV and ZIKV, and 10^7^ FFU/mL for DENV-1 and confirmed by titration of blood aliquots collected after the feeding experiment. After the infectious blood meal, fully engorged female mosquitoes were transferred to 1.2 L containers covered with netting and maintained in a climatic chamber (Memmert, Schwabach, Germany) at 28°±1°C, 16h:8h light:dark cycle and 80% humidity. Ten percent sucrose was provided as a nutrient source.

### Infection, dissemination, and transmission analysis

For each population, batches of 19–34 mosquitoes were analyzed at 7 and 14 days post-exposure (dpe). Additionally, for CHIKV, mosquitoes were evaluated at 3 dpe. These time points were selected according to the replication kinetics of DENV, CHIKV, and ZIKV in mosquitoes [15–19]. Each mosquito was processed as follows: abdomen-thorax were examined to estimate the viral infection, the head was used to assess viral dissemination beyond the mosquito midgut, and saliva was collected from individual mosquitoes as described in Dubrulle et al. [15] to determine transmission. Briefly, for each mosquito, wings and legs were removed and the proboscis was inserted into a 20 μL pipette tip containing 5 μL of fetal bovine serum (FBS). After 30 min, FBS containing saliva was expelled in 45 μL of culture media (Leibovitz L15 for DENV-1, Dulbecco’s modification of Eagle’s medium (DMEM) for ZIKV and CHIKV) for titration. Additionally, heads were removed from thorax-abdomen and both components were separately homogenized in 300 μL of culture media supplemented with 2% FBS. Then, the homogenates were centrifuged at 10000x*g* for 5 min and the supernatants stored at -80 °C until titration.

### Viral titration

For ZIKV and CHIKV, thorax-abdomen and head homogenates were serially diluted and inoculated onto monolayers of Vero cells in triplicate in 96-well plates. Cells were incubated for 7 days for ZIKV and 3 days for CHIKV at 37°C, then stained with a solution of crystal violet (0.2% in 10% formaldehyde and 20% ethanol). Evidence of viral particles was assessed by detection of CPE. Saliva was titrated on monolayers of Vero cells in 6 well plates, and incubated for 7 days for ZIKV and 3 days for CHIKV under an agarose overlay. Titers of saliva were expressed as pfu (plaque-forming unit)/saliva.

For DENV, thorax-abdomen samples, head homogenates and serially diluted saliva were inoculated into C6/36 cells in 96-well plates. After incubation at 28°C for 5 days, the plates were stained using hyper-immune ascetic fluid specific to DENV as primary antibody (Merck). Alexa Fluor 488 goat anti-mouse IgG was used as the second antibody (Life Technologies, Carlsbad, USA).

Infection rate (IR) corresponds to the proportion of mosquitoes with infected bodies (abdomen-thorax) among those tested, while dissemination rate (DR) refers to the proportion of mosquitoes with virus detected in heads among those tested. Ultimately, transmission rate (TR) represents the proportion of mosquitoes with infectious saliva among the total number of mosquitoes tested (i.e., surviving females including females not infected and unable to disseminate the virus and those able to disseminate). Saliva titers were also estimated, and means ±SEs were calculated for all positive saliva samples.

### Statistical analysis

All statistical tests were conducted using R V. 3.3.2 [20]. For each virus, the different rates (IR, DR, and TR) were compared between the two mosquito populations and the different dpe. Within each dpe, the rates of the three viruses were compared, taking into consideration the mosquito population. All these comparisons were performed with Fischer’s exact tests. When necessary, multiple Fisher’s exact tests were applied (package “rcompanion”) with an adjustment of the significance level by the sequential Bonferroni method. The variances of the different rates were calculated with the binomial proportion confidence intervals at 95%. For each virus, the saliva titers were compared between mosquito populations using Wilcoxon tests. Because of the low virus-positive saliva detected, these results were all statistically non-significant. All comparisons with *P* values > 0.05 were considered non-significant.

## Results

### Transmission rates of Cuban *Ae*. *aegypti* populations were low for DENV-1 and ZIKV

**Fig 2:**
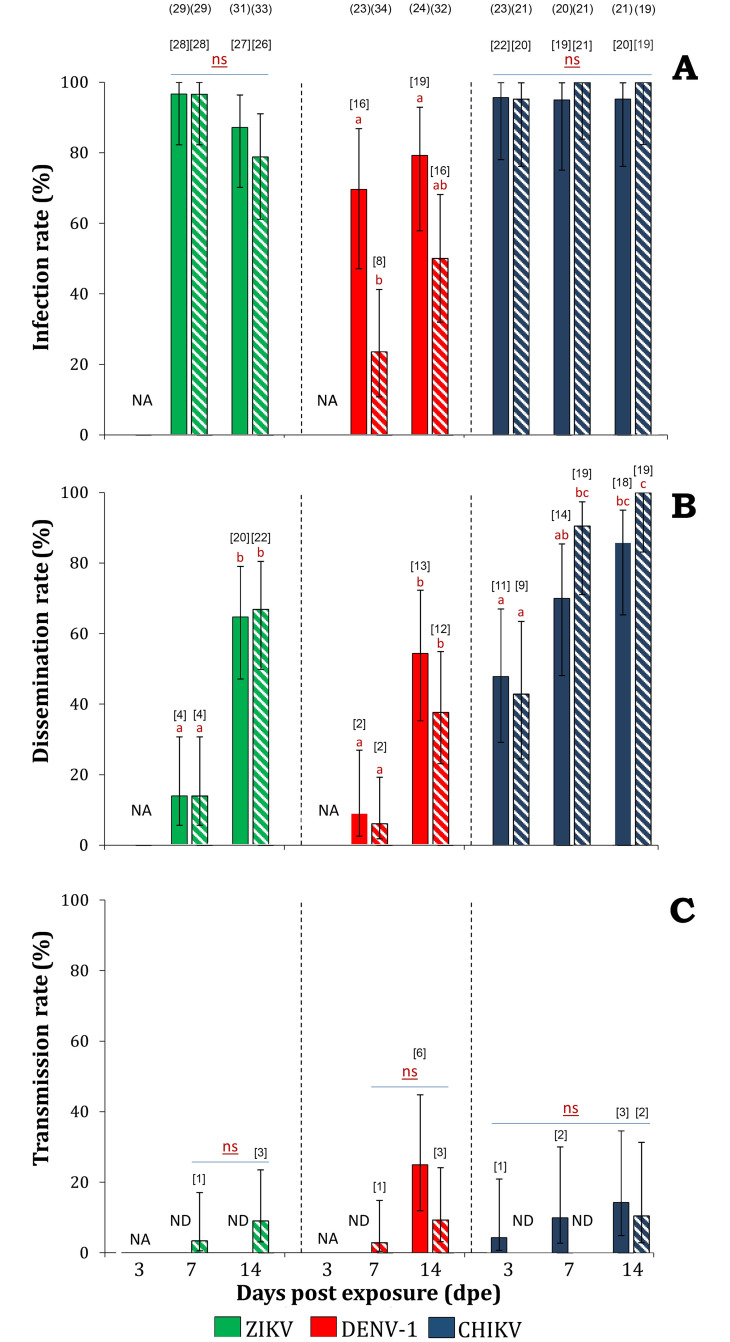
Infection (A), dissemination (B), and transmission (C) rates (± 95% CI) of two *Aedes aegypti* populations from Havana, Cuba, at days 3, 7, and 14 after exposure to mono-infected blood meals containing Zika (ZIKV), dengue-1 (DENV-1), or chikungunya (CHIKV) viruses. Pasteur population (PTE) is represented with filled bars and Párraga (PRG) with stripped bars. ND: Not detected, NA: Not assayed, ns: non-significant (*P* > 0.05). Different superscript letters indicate significant differences (*P* < 0.05) between populations and/or dpe for a given virus. Number of individuals tested at each mosquito populations/virus/dpe are given in parenthesis. Numbers in brackets above each column indicate the positive samples for each combination of mosquito population/virus/dpe.

To assess the vector competence of *Ae*. *aegypti* from Havana for the main flaviviruses that have circulated in Cuba, PTE and PRG populations were artificially monoinfected with DENV-1 and ZIKV and their IRs, DRs, TRs, and saliva titers were estimated at 7 and 14 dpe for the two viruses (Fig 2, Table 1). Susceptibility for DENV-1 and ZIKV infection was observed in the two mosquito populations, but overall, they were less susceptible to DENV-1 (IRs range: 24–79%) when compared to ZIKV infection (IRs range: 79–97%), especially at 7dpe (*P* = 0.03 for PTE; *P* < 0.0001 for PRG). No statistical differences were detected among mosquito populations nor dpe for ZIKV, whereas for DENV-1, the mean IRs for PRG were lower (37%) than that of PTE population (74%), which was statistically significant at 7dpe (*P* = 0.003). Dissemination rates ranged from 14–67% for ZIKV and from 6–54% for DENV-1. No statistical differences of DRs were detected between viruses nor mosquito populations at the same dpe. However, DRs increased at 14 dpe for both viruses regardless of the mosquito population (*P* < 0.05 for all populations/viruses combinations).

**Table 1:** Mean viral (± SE) loads in the saliva of two *Aedes aegypti* populations: Pasteur (PTE) and Párraga (PRG) from Havana, Cuba, at days 3, 7, and 14 after exposure to mono-infected blood meals containing, Zika (ZIKV), dengue-1 (DENV-1), or chikungunya (CHIKV) viruses.

Virus	PTE Log_10_ titre (pfu/saliva)*						PRG Log_10_ titre (pfu/saliva)*					
	3dpe		7dpe		14dpe		3dpe		7dpe		14dpe	
	Mean	±SE	Mean	±SE	Mean	±SE	Mean	±SE	Mean	±SE	Mean	±SE
ZIKV	NA	-	ND	-	ND	-	NA	-	0.5 (1)	Nap	0.5 (3)	±0.2
DENV-1	NA	-	ND	-	0.6 (6)	±0.2	NA	-	1.2 (1)	Nap	0.7 (3)	±0.3
CHIKV	0.2 (1)	Nap	0.6 (2)	±0.4	1.4 (3)	±0.6	ND	-	ND	-	0.9 (2)	±0.4

Numbers of positive saliva samples in parenthesis. ND: Not detected, NA: not assayed, Nap: Not applicable, dpe: days post-exposure.

*For DENV-1, virus titer is expressed in focus forming unit (FFU)/saliva.

Regarding transmission potential, low TRs were observed for both viruses regardless of the mosquito population. No ZIKV particles were detected in saliva of the examined mosquitoes from PTE population, whereas the virus was detected as early as 7 dpe in the saliva of PRG mosquitoes (TR_PRG/ZIKV_ range 4–9%). For DENV-1, the virus was detected in the saliva of PRG population at 7 dpe (TR = 3%) but was not detected for PTE until 14 dpe. At this time point, the TR recorded for PTE (25%) and for PRG population (9%) were not statistically different (P = 0.15) (Fig 2). Similarly, no statistical differences were observed in TRs nor viral loads between ZIKV and DENV-1 at any dpe. The mean viral loads detected in saliva from PRG population infected with DENV-1 at days 7 and 14 pe was 0.8±0.3 log_10_ FFU/saliva, while that of ZIKV was 0.5±0.1 log_10_ pfu/saliva (Table 1).

### *Ae*. *aegypti* from Cuba were able to transmit CHIKV at 3dpe

The ability of PTE and PRG to become infected with and transmit CHIKV was evaluated at 3, 7, and 14 dpe (Fig 2). Both populations were highly susceptible to CHIKV infection with IRs ranging between 95–100% at 3 dpe. Dissemination was also detected at 3 dpe and then significantly increased at 7 and 14 dpe in both mosquito populations (Fig 2). At 14 dpe, the estimated DRs for PTE and PRG populations were 86 and 100%, respectively. Conversely, the transmission potential was low for both populations, with similar TRs in PTE (range 4–14%) and PRG (range 0–11%) (Fig 2). Mosquitoes from PTE population were able to expectorate virus at 3 dpe, whereas virus was first detected in saliva from PRG at 14 dpe. The mean CHIKV load measured in *Ae*. *aegypti* saliva for PRG population ranged from 0–0.9 log_10_ pfu/saliva, whereas in mosquitoes from PTE, the mean viral loads ranged from 0.2–1.4 log_10_ pfu/saliva and reached a maximum at 14 dpe (Table 1).

## Discussion

Although mosquito species implicated in virus transmission have been historically studied in Cuba [21,22], their intrinsic ability to transmit DENV, ZIKV, and CHIKV has never been explored until now. Here, we confirm for the first time that *Ae*. *aegypti* from Cuba is able to transmit these arboviruses of major medical importance, albeit at low levels.

Although we failed to detect ZIKV in the saliva of PTE population, our results showed that *Ae*. *aegypti* from PRG were able to transmit the flaviviruses DENV-1 and ZIKV at 7 dpe supporting their involvement in recent epidemics in Cuba [2]. Despite the susceptibility of *Ae*. *aegypti* to the infection by these viruses, the TRs obtained at 7 and 14 dpe for DENV-1 and ZIKV were low. Previous works assaying vector competence of *Ae*. *aegypti* populations from different parts of the world with ZIKV Asian strains also have highlighted a poor transmission potential in this species [18,23–25], which contrasts with the rapid ZIKV spread across the American territories. The large number of naïve human populations for ZIKV has been suggested as an explanation to balance the low transmission potential of *Ae*. *aegypti* populations for this virus [23]. Given the number of DENVs outbreaks and epidemics that have taken place in Cuba (reviewed in [2]), it was also expected to find higher TRs with the DENV-1 strain assayed. However, DENVs also include other serotypes and the vector competence of Cuban populations for these may differ as seen elsewhere [26,27]. Hence, further studies should be conducted to estimate the vector competence of Cuban mosquitoes to the other DENV serotypes.

The other *Aedes*-borne virus that has caused epidemics in the Americas in recent years is CHIKV. Curiously, although this alphavirus was actively circulating in the region, no CHIKV autochthonous transmission has been recorded in Havana so far despite the importation of chikungunya cases into the island [2]. Here, we demonstrated that *Ae*. *aegypti* populations from Havana were able to transmit CHIKV (albeit at low TRs) as early as 3 days after ingesting virus (in the case of PTE population), suggesting that the absence of local circulation is not linked to the refractoriness of local vectors to this alphavirus. Such short EIP challenges the implementation of vector control strategies, favors the rapid propagation of primary autochthonous cases, and could lead to explosive epidemics. Indeed, the EIP is considered a critical factor in determining the efficiency of transmission by a vector [28,29].

It is important to point out that arbovirus transmission in nature cannot be exclusively assessed by vector competence parameters. The contribution of vector competence to the mosquito vectorial capacity, although essential, is weaker when compared to other ecological traits (i.e. anthropophagy, survivorship, numbers of bites) [28]. Therefore, the influence of these latter factors may offset the low vector competence of Cuban *Ae*. *aegypti* populations for the tested arbovirus and enhance their vectorial capacity, which could explain why "poorly efficient vectors" can trigger epidemics [30]. In Cuba, despite efforts to control vector populations, *Ae*. *aegypti* is one of the most widespread and abundant mosquito species [14]. Such spread and abundance may counteract the rates of transmission detected, facilitating DENV, ZIKV, and CHIKV epidemics.

The experimental design and the techniques used may also impact vector competence estimations in laboratory assays. Artificial feeding systems (like the one used in the present study) may underestimate the levels of vector competence, because they are less efficient in ensuring mosquito infection and dissemination when compared to the use of animal models [18,31]. For this reason, we used virus titers in the artificial blood-meal (10^7^ TCID_50_/mL for CHIKV and ZIKV, and 10^7^ FFU/mL for DENV-1) higher than typical viremias observed in natural hosts [18,31–33]. In addition, it has been shown that long-term freezing viruses (like used here) may yield significantly lower mosquito infections than the same titer of freshly harvested virus (reviewed by [34]). Furthermore, *in vitro* saliva collection methods may underestimate the amount of virus deposited during *in vivo* feeding [35–37] and the sensitivity of virus titration using cell culture assays is lower than the virus inoculation in mice, for example, as demonstrated by Smith et al., 2005 [36]. Finally, it is probably that DRs and TRs for the tested flaviviruses are higher at later day post exposure as reported elsewhere (i.e. 21dpe) [17,24], but this day point was not assessed in our study as *Ae*. *aegypti* life expectancy in the field is generally shorter (~3–7 days) [38]. All these factors could be related with the low transmission potential observed rather than actual intrinsic factors within the mosquito populations.

In the present study, both vector populations exhibited high IRs and DRs for ZIKV (IR>79% and DR>65% at 14 dpe) and CHIKV (IR>95% and DR>86% at 14 dpe), similar to previous reports from the Americas, suggesting elevated permeability of the midgut infection and escape barriers [16,23,25,39,40]. Conversely, both populations were less susceptible to DENV-1 infection when compared to the other viruses, highlighting a higher efficiency of the midgut barrier and/or mosquito immunity in preventing DENV-1 replication [41,42]. Regarding transmission, the low TRs observed overall suggest that salivary gland barriers play the most important role in limiting the expectoration of virions, as it has been proposed previously [16,25,39–41,43,44]. This phenomenon highlights the importance of assessing transmission in vector competence assays, since well-disseminated infections do not necessarily result in high transmission rates.

The low transmission potential observed in the mosquito populations examined could lead to suspicion of other vectors besides *Ae*. *aegypti*. *Ae*. *albopictus* (registered in Cuba since 1995 [45]) may be involved in the natural transmission of these flaviviruses, as reported elsewhere (reviewed in [46]). Although vector competence of local populations of this mosquito have never been explored, it is less probable that *Ae*. *albopictus* from Havana play a significant role in the transmission of these arboviruses because this species is more of a generalist feeder than *Ae*. *aegypti* [47] and so far it is confined to peri-urban settings, which limits its contact with large human populations [14]. So, it is less likely for *Ae*. *albopictus* than *Ae*. *aegypti* to take at least two separate blood meals from a human, allowing for acquisition and transmission of an arbovirus [48].

In light of our experimental evidence, where the same vector can transmit (with similar ability) the three assayed arboviruses, one might wonder why the recent CHIKV emergence did not lead to outbreaks in Havana, while that of ZIKV led to epidemics throughout the country? The active surveillance and quarantine of imported symptomatic cases could have played an important role in this epidemiological scenario, given the lesser asymptomatic proportion of CHIKV (4–28%) [49] when compared to that of ZIKV (<80%) [50]). Also, undetermined entomological or ecological reasons could have hampered the spread of the virus in the island as it has been seen before 2013 in other territories (reviewed by [49]). Another hypothesis would be a hidden circulation or misdiagnosed CHIKV infections, but in view of the higher proportion of CHIKV infected persons with clinical symptoms requiring medical attention when compared to other arboviral infections [51], such explanation is less plausible.

In conclusion, the vector competence assessment conducted on *Ae*. *aegypti* populations from Havana demonstrated the ability of this species to serve as a vector of DENV, ZIKV, and CHIKV in Cuba. These results, along with the widespread distribution and high abundance of this species in the urban settings throughout the island, underline the importance of *Ae*. *aegypti* control and arbovirus surveillance to prevent future outbreaks.
